# Bioactivity of Nonedible Parts of *Punica granatum* L.: A Potential Source of Functional Ingredients

**DOI:** 10.1155/2013/602312

**Published:** 2013-07-08

**Authors:** Nawraj Rummun, Jhoti Somanah, Srishti Ramsaha, Theeshan Bahorun, Vidushi S. Neergheen-Bhujun

**Affiliations:** ^1^Department of Health Sciences, Faculty of Science, University of Mauritius, Réduit, Mauritius; ^2^Department of Biosciences, and ANDI Centre of Excellence for Biomedical and Biomaterials Research, University of Mauritius, Réduit, Mauritius; ^3^Department of Health Sciences, Faculty of Science and ANDI Centre of Excellence for Biomedical and Biomaterials Research, University of Mauritius, Réduit, Mauritius; ^4^ANDI Centre of Excellence for Biomedical and Biomaterials Research, University of Mauritius, Réduit, Mauritius

## Abstract

*Punica granatum* L. has a long standing culinary and medicinal traditional use in Mauritius. This prompted a comparative study to determine the bioefficacy of the flower, peel, leaf, stem, and seed extracts of the Mauritian *P. granatum*. The flower and peel extracts resulting from organic solvent extraction exhibited strong antioxidant activities which correlated with the high levels of total phenolics, flavonoids, and proanthocyanidins. The peel extract had the most potent scavenging capacity reflected by high Trolox equivalent antioxidant capacity value (5206.01 ± 578.48 *μ*mol/g air dry weight), very low IC_50_ values for hypochlorous acid (0.004 ± 0.001 mg air dry weight/mL), and hydroxyl radicals scavenging (0.111 ± 0.001 mg air dry weight/mL). Peel extracts also significantly inhibited *S. mutans* (*P* < 0.001), *S. mitis* (*P* < 0.001), and *L. acidophilus* (*P* < 0.05) growth compared to ciprofloxacin. The flower extract exhibited high ferric reducing, nitric oxide scavenging, and iron (II) ions chelation and significantly inhibited microsomal lipid peroxidation. Furthermore, it showed a dose-dependent inhibition of xanthine oxidase with an IC_50_ value of 0.058 ± 0.011 mg air dry weight/mL. This study showed that nonedible parts of cultivated pomegranates, that are generally discarded, are bioactive in multiassay systems thereby suggesting their potential use as natural prophylactics and in food applications.

## 1. Introduction

**Figure 1:**
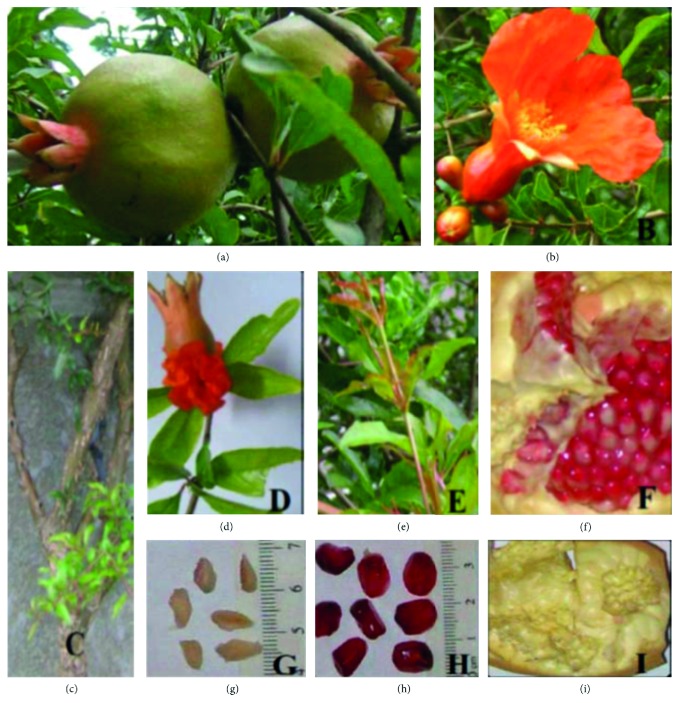
Different anatomical parts of *P. granatum* tree and fruit. (a) Unripe fruit; (b) flower; (c) stem; (d) flower and tubular calyx; (e) young leaves at the branch endings; (f) the fruit's rind with membranous extensions forming compartments which contain the juicy arils; (g) seeds; (h) arils (juicy pulp coating the seed); (i) pomegranate inner membrane.

*Punica granatum* L. fruit or fruit juice has for the past decade been advocated as an interesting functional food that can confer health benefits beyond basic nutrition [1, 2]. *P. granatum* L. belongs to the family of Punicaceae and is indigenous to the Himalayas in northern India and to Iran [3] but has grown and been naturalized in a number of Asian and African countries including Mauritius. The edible and nonedible parts (Figure 1) have been reported to treat different pathological conditions in different folklore medicine [4–6]. Documented use of pomegranate in Mauritian folklore medicine includes ingestion of macerated bark extracts to treat asthma, chronic diarrhea, chronic dysentery, relaxation of the larynx, and intestinal worms [7]. 

Pomegranate extracts are known for their antidiabetic, antibacterial, anticarcinogenic, antiatherogenic, and antihypertensive potential amongst others [3]. Pomegranate juice is also used as mouthwash in oral hygiene [8]. Consumption of pomegranate juice has been linked with a decrease in inflammatory biomarkers levels and oxidation of both proteins and lipids in a randomized placebo-controlled trial [9]. In the same vein, the beneficial effect of pomegranate juice was reported in an initial phase II clinical trial in patients with prostate cancer [10]. The health benefits of pomegranate have been ascribed to the pluripharmacological effects of the secondary metabolites more specifically its polyphenolic compounds present in relatively high concentrations [11–13]. 

The phytophenolic compositions vary differently in the edible and nonedible parts of the plants and have been widely investigated. Pomegranate fruit (peel, aril, seeds, and juice) has been reported to be rich in phenolic acids, flavanols, flavones, flavonones, anthocyanidins, and anthocyanin [3]. Literature data reported glycated anthocyanins (pelargonidin 3,5-diglucoside, pelargonidin 3-glucoside) apart from phenolic compounds common to the edible parts like gallic acid in the flowers [14], while the nonedible parts of pomegranate comprising leaves, roots, and stem contained apigenin, punicalin, punicalagin, and luteolin [3, 15, 16]. This rich polyphenolic composition has been intrinsically linked to the pluripharmacological effects of pomegranate extracts. However, it should be noted that sources of variation in the level of phytochemicals and nutrients arising from genetic variability of a naturalized plant in addition to geographical and environmental factors can result in diverse polyphenolic compositions that modulate bioactivity level.

Whilst recent years have witnessed a surge in the scientific evaluation of the ethnopharmacological uses of pomegranate, limited works have been reported on the assessment of the nonedible discarded parts as a source of bioactive ingredients for the functional food industry. Thus, this study aimed at determining the antibacterial, anti-inflammatory, and antioxidant potential of the nonedible parts of the Mauritian cultivar of pomegranate with the view of promoting their utilization in functional health and in potential food applications. 

## 2. Methodology

### 2.1. Chemicals and Microorganisms

*Streptococcus mutans* (ATCC^R^ 5175), *Streptococcus mitis* (ATCC^R^ 6249), and *Lactobacillus acidophilus* (ATCC^R^ 4356) were purchased from ATCC. Brain heart infusion agar (BHI) and de Man, Rogosa and Sharpe agar (MRS) were purchased from Sigma-Aldrich, Germany. BHI was used for growth of *S. mutans* and *S. mitis* while MRS agar was utilized for *L. acidophilus*. All other chemicals used were of analytical grade.

### 2.2. Plant Materials

Pomegranate plant and fruit parts were collected from a domesticated plant growing in a backyard in “Triolet” village situated in the Pamplemousses district in the northern part of Mauritius Island, during the month of September 2011 and authenticated by the herbarium of Mauritius Sugar Industry Research Institute, Réduit. *Punica granatum *leaves, stems, flowers, and fruits were collected from the same plant. The latter were air dried and the samples homogenized to a fine powder prior to extraction. 

### 2.3. Preparation of Extracts

The plant material was extracted thrice with 70% methanol (1 : 3, w/v) and allowed to macerate each time at 4°C for 24 hours. The filtrates were pooled together and concentrated *in vacuo* at 37°C. The concentrated aqueous extract was partitioned in dichloromethane to remove fats and chlorophyll, and the aqueous phase was then collected and lyophilized. The lyophilized powders, thereof derived, were dissolved in deionized water and in 100% methanol to a concentration of 1 g of air dried mass to 5 mL for the subsequent tests.

### 2.4. Total Phenolic Content (TPC)

The total phenolic content was estimated using the Folin-Ciocalteu assay adapted from Neergheen et al. [17]. The reaction mixture in a final volume of 5 mL contained 0.25 mL of the extracts, 3.50 mL of distilled water, and 0.25 mL of Folin-Ciocalteu reagent. After 3 minutes, 0.75 mL of 20% sodium carbonate solution was added. The tubes were mixed thoroughly and heated for 40 minutes in a water-bath set at 40°C and then allowed to cool. The absorbance of the blue coloration was read at 685 nm against a blank. Total phenolics were calculated with respect to a gallic acid standard curve (stock solution 250 *μ*g/mL) and results expressed in mg of gallic acid equivalent (GAE)/g air dry weight (ADW) of plant material.

### 2.5. Total Proanthocyanidin Content (TPrC)

A modified HCl/Butan-1-ol assay adapted from Porter et al. [18] was used for the quantification of total proanthocyanidin content of the methanolic plant extracts. The reaction mixture in each tube contained, in a final volume of 3.35 mL, the following in order of addition: 0.25 mL extract, 3 mL of n-BuOH/HCl (95 : 5 v/v), and 0.1 mL of 2% NH_4_Fe (SO_4_)_2_·12 H_2_O in 2 M HCl, and the tubes were incubated for 40 minutes at 95°C. A red coloration was developed, and the absorbance was read at 550 nm against a blank standard containing 0.25 mL n-BuOH/HCl (95 : 5 v/v) instead of extract. The amount of proanthocyanidins in the extracts was calculated, in triplicates, with respect to a cyanidin chloride standard curve (stock solution 0.1 mg/mL). Results were expressed in mg of cyanidin chloride equivalent (CCE)/g ADW of plant material.

### 2.6. Total Flavonoid Content (TFC)

Total flavonoids were measured using a colorimetric assay adapted from Zhishen et al. [19]. A total of 150 *μ*L of 5% aqueous NaNO_2_ was added to 2.50 mL of extract. After 5 min, 150 *μ*L of 10% aqueous AlCl_3_ was added. A total of 1 mL of 1 M NaOH was added 1 min after the addition of aluminum chloride. The absorbance of the solution was measured at 510 nm. Flavonoid contents were expressed in *μ*g quercetin/g of ADW of plant material.

### 2.7. Ferric Reducing Antioxidant Power Assay (FRAP)

The reducing power of the extracts was assessed using the method of Benzie and Strain [20]. A total of 100 *μ*L of sample was added to 300 *μ*L of distilled water, followed by 3 mL of FRAP reagent (40 mM HCl and 20 mL of 20 mM ferric chloride in 200 mL of 0.25 M sodium acetate buffer at pH 3.6). The absorbance was read at 593 nm after 4 min of incubation at 37°C. Results were expressed in *μ*mol Fe^2+^ equivalent/g of ADW of plant material.

### 2.8. Trolox Equivalent Antioxidant Capacity (TEAC) Assay

The free radical scavenging capacity of the extracts was measured by the TEAC assay according to the method of Campos and Lissi [21]. A total of 0.50 mL of diluted plant extract was added to 3 mL of the ABTS^•+^ solution generated by a reaction between 2,2-azino-bis(3-ethyl-benzthiazoline-6-sulfonic acid) diammonium salt (ABTS, 0.50 mM) and activated MnO_2_ (1 mM) in phosphate buffer (0.10 M, pH 7). Decay in absorbance was monitored at 734 nm for 15 min. TEAC values are expressed in *μ*mol Trolox equivalent/g of ADW.

### 2.9. Iron (II) Chelating Activity

The method of Neergheen et al. [17] was adapted to assess the iron (II) chelating activity of the extracts. The reaction mixture contained, in order of addition, 200 *μ*L of plant extract (varied concentrations) and 50 *μ*L of FeCl_2_·4H_2_O (0.5 mM). The reaction volume was made up to 1 mL with distilled deionised water and incubated for 5 minutes at room temperature. After incubation, 50 *μ*L of FerroZine (2.5 mM) was added and the purple coloration formed was read at 562 nm. The absorbance of the reaction mixture was read both before and after the addition of FerroZine to account for possible interferences caused by the plant extract. The controls contained all the reaction reagents and water instead of the extract or the positive control substance. EDTA was used as a positive control. The percentage chelating activity was calculated and results were expressed as mean IC_50_ (mg ADW/mL). 

### 2.10. Scavenging of Hypochlorous Acid (HOCl)

The ability of the extracts to scavenge HOCl was assessed essentially as described by Neergheen et al. [17]. Briefly, the reaction mixture contained 100 *μ*L taurine (10 mM), 100 *μ*L HOCl (1 mM), 100 *μ*L plant extract (variable concentrations), and 700 *μ*L phosphate saline buffer (pH 7.4) in a final volume of 1 mL. The solution was mixed thoroughly and incubated for 10 minutes at ambient temperature. The sample was then assayed for taurine chloramine by adding 10 *μ*L potassium iodide to the reacting mixture. The I_2_ released was determined spectrophotometrically at 350 nm in the presence of excess I^−^ as I_3_^−^. The absorbance of the reaction mixture was read both before and after the addition of potassium iodide as to cater for possible interferences caused by the plant extract. The analyses were made in triplicates and the results were expressed as IC_50_ mg ADW/mL for the plant extracts.

### 2.11. Inhibition of Deoxyribose Degradation

The hydroxyl radical scavenging potential of the extracts was determined using the deoxyribose assay [22]. The reacting mixture contained in a final volume of 1 mL the following reagents, order of addition indicated: 200 *μ*L of 100 mM KH_2_PO_4_-KOH, 200 *μ*L of 0.5 mM FeCl_3_, 100 *μ*L of 1 mM EDTA, 100 *μ*L sample, 200 *μ*L of 15 mM deoxyribose, 100 *μ*L of 10 mM H_2_O_2_, and 100 *μ*L of 1 mM ascorbic acid. Reaction mixtures were incubated at 37°C for 1 hour. 

At the end of the incubation period, 1 mL 1% (w/v) thiobarbituric acid (TBA) was added to each mixture followed by the addition of 1 mL 2.8% (w/v) trichloroacetic acid (TCA). The solutions were heated in a water bath at 80°C for 20 min to develop the pink coloured MDA-(TBA)_2_ adduct. The MDA-(TBA)_2_ chromogen was extracted into 3 mL butan-1-ol and its absorbance measured at 532 nm. The analyses were made in triplicates and the results were expressed as IC_50 _g ADW/mL for the plant extracts. 

### 2.12. Inhibition of Microsomal Lipid Peroxidation

Beef liver microsomes were prepared by tissue homogenization as described by Neergheen et al. [17]. The formation of malondialdehyde, measured as thiobarbituric reactive substances (TBARS), was used to monitor microsomal lipid peroxidation. The reaction mixture contained in a final volume of 1 mL the following: 200 *μ*L of 3.4 mM phosphate buffered saline (pH 7.4), 200 *μ*L of 0.5 mg/mL microsomal protein, 400 *μ*L of sample (variable concentrations), 100 *μ*L of 1 mM FeCl_3_, and 100 *μ*L of 1 mM ascorbate. The mixture was incubated for 1 hour at 37°C.

At the end of the incubation period, 100 *μ*L 2% (w/v) BHT was added followed by 1 mL 1% (w/v) thiobarbituric acid and 2.8% (w/v) trichloroacetic acid. The solutions were heated in a water bath at 80°C for 20 min to develop the pink coloured MDA-(TBA)_2_ adduct. As turbidity was encountered, the MDA-(TBA)_2_ chromogen was extracted into 2 mL butan-1-ol and its absorbance measured at 532 nm. The inhibition of microsomal lipid peroxidation was calculated and results are expressed as mean IC_50_ (mg ADW/mL).

### 2.13. Superoxide Anion Radical Scavenging Assay

The superoxide anion scavenging activity of the pomegranate extracts was measured according to the modified method of Kumar et al. [23]. One mL of 156 *μ*M of nitroblue tetrazolium (NBT) aqueous solution and 1 mL of 200 *μ*M beta-nicotinamide adenine dinucleotide reduced disodium salt hydrate (NADH) aqueous solution were mixed together, followed by the addition of 1 mL of aqueous pomegranate extract (varied concentration). The reaction was started by adding 100 *μ*L of 60 *μ*M phenazine methosulphate (PMS) aqueous solution. The reaction mixture was incubated at 25°C for 20 minutes and the absorbance was measured at 560 nm against control sample. Ascorbic acid was used as a positive control. IC_50_ value was calculated from the dose-dependent curve obtained by plotting antioxidant activity (%) against a concentration range for each pomegranate extract. The antioxidant activity was calculated as follows:

(1) $$\begin{array}{c} \text{antioxidant}\;\text{activity}\;\%=\left[\frac{\left(A_{0}-A_{1}\right)}{A_{0}}\right]\times 100, \end{array}$$

where *A*_0_ is the absorbance of the control (reaction mixture without test sample), and *A*_1_ is the absorbance of the test sample. 

### 2.14. Nitric Oxide Radical Inhibition Assay

Nitric oxide radical inhibition was evaluated according to the modified method of Sunil et al. [24]. Griess Illosvoy' reagent was modified by using 0.1% (w/v) naphthylethylenediamine dihydrochloride. The reaction mixture contained 0.5 mL of extracts (variable concentrations), 2 mL of 10 mM aqueous sodium nitroprusside, and 0.5 mL phosphate saline buffer. The mixture was incubated at 25°C for 180 minutes. 0.5 mL of the reaction mixture was pipetted out, and 2 mL of Griess Illosvoy's reagent (0.33% sulphanilic acid in 20% glacial acetic acid and 0.1% naphthylethylenediamine dichloride) was added, mixe,d and allowed to stand for 30 minutes. The absorbance of the pink chromophore formed was measured at 546 nm. Ascorbic acid was used as positive control and the percentage antioxidant activity was calculated. IC_50_ value was calculated from the dose-dependent curve obtained by plotting antioxidant activity (%) against a concentration range for each pomegranate extract.

### 2.15. Xanthine Oxidase (XO) Inhibition Assay

Spectrophotometric determination of XO inhibitory activity measuring uric acid production from xanthine substrate was used. The method was adapted from Havlik et al. [25] with some modifications. The mixture consisted of 250 *μ*L extract (varied concentrations), 400 *μ*L 0.12 M phosphate buffer (pH 7.5), and 330 *μ*L xanthine (8 mM in same buffer). The reaction was initiated by adding 20 *μ*L of xanthine oxidase (0.5 U/mL in same buffer) which was prepared immediately before use. The tubes were incubated at room temperature for 5 minutes and the reaction stopped by the addition of 200 *μ*L 1 M HCl. Absorbance for formation of uric acid was read at 295 nm. Allopurinol was used as the positive control (concentration range: 25–350 *μ*M). The % inhibition was calculated and activity of extract presented as calculated IC_50_ (*μ*M).

### 2.16. Determination of Minimum Inhibition Concentration of *Punica granatum*

Sterilized molten agar was dispensed into sterile Petri dishes and allowed to solidify. Microbial suspension (150 *μ*L) containing approximately 1.5 × 10^8^ CFU/mL was spread evenly over the surface of the solidified medium and left to air dry. Meanwhile, 20 *μ*L of sample extracts and ciprofloxacin (2 mg/mL) were loaded separately onto sterile oven-dried paper discs and placed firmly onto medium using forceps. Each plate consisted of four impregnated discs: two extracts, one positive control, and one extract + positive control. The experiment was performed in quadruplicate.

The Petri dishes were inverted and incubated at 37°C for 24 hours. After the incubation period, the diameter of the zone of inhibition, defined as the area which was devoid of or had minimal cell growth, was measured to the nearest millimeter. The antimicrobial activity of the extract was determined by the zone of inhibition of the extracts; a higher inhibition zone indicated a more potent antimicrobial effect of the extract.

### 2.17. Statistical Analysis

All the antioxidant assays were carried out in triplicate and the results recorded were expressed as mean ± standard deviation. All charts including standard curves, dose response curves, and bar charts were generated using Microsoft Excel software (Version 2010) and GraphPad Prism, version 6.01, from GraphPad Software (San Diego, CA, USA). Correlation between phytoconstituent and antioxidant activity was carried out using the Pearson correlation on SPSS (version 17.0). ANOVA (single factor) was performed in Microsoft Excel software (Version 2010) to test for significant difference in mean values of the different extracts for each assay. To test for null hypothesis, the least significant difference between extracts, for each independent assay, was calculated.

## 3. Results

### 3.1. Polyphenolic Content

Total phenolics of the extracts ranged between 0.65 ± 0.004 mg GAE/g ADW and 336.51 ± 0.70 mg GAE/g ADW with the highest content measured in the flower (Table 1). The amount of total phenolics differed significantly between the extracts (*P* < 0.05). Total flavonoids were between 0.332 ± 0.003 mg QE/g ADW and 213.54 ± 3.14 mg QE/g ADW. Pomegranate flower extract had the most prominent flavonoid level followed by peel, leaf, and stem, with only negligible amount measured in the seed extract. Significant differences were observed in flavonoid content among the extracts (*P* < 0.05). Relatively lower amount of total proanthocyanidins was present in the samples compared to the amount of total phenolics and flavonoid contents (Table 1). 

### 3.2. Antioxidant Activities

**Figure 2:**
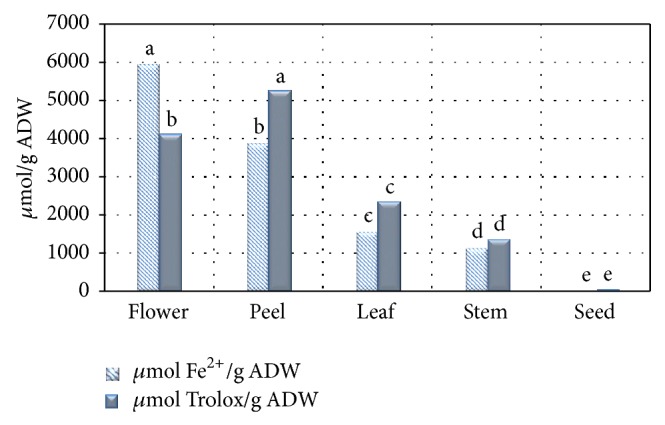
TEAC and FRAP values of different pomegranate extracts. ADW: air dry weight. Different superscripts between columns represent significant difference between extracts. Data expressed as mean ± standard deviation *μ*mol Trolox equivalent/g air dry weight for TEAC (*n* = 3); LSD = 920.44, at 5% significance. Data expressed as mean ± standard deviation *μ*mol Fe^2+^/g ADW for FRAP (*n* = 3); LSD = 101.34, at 5% significance.

TEAC value ranged from 14.04 ± 2.40 *μ*mol Trolox/g ADW to 5206.01 ± 578.48 *μ*mol Trolox/g ADW with the peel exhibiting the highest TEAC value. The ferric reducing potential ranged between 6.29 ± 0.38 *μ*mol Fe^2+^/g ADW and 5933.00 ± 54.06 *μ*mol Fe^2+^/g ADW (*P* < 0.05) (Figure 2). The flowers had the highest ferric reducing potential which was statistically different from the activity of the other extracts (*P* < 0.05) (Figure 2).

All extracts showed dose-dependent iron (II) chelating activity. However, pomegranate flower exhibited the highest iron (II) cation chelating activity with the lowest calculated IC_50_ value (Table 2). Statistically significant differences were observed between the IC_50_ values (*P* < 0.05) with pomegranate flower, peel, and stem being more potent than the leaf and the seed. Similarly, most of the extracts except the stem extract showed dose-dependent hypochlorous acid scavenging activity with the peel extract being the most potent HOCl scavenger. The calculated IC_50_ value ranged between 0.004 ± 0.001 mg ADW/mL and 5.200 ± 0.400 mg ADW/mL (Table 2). The calculated IC_50_ value of flower, peel, and leaf extracts differed significantly from that of the seed (*P* < 0.05). However, the flower, peel, and leaf were observed to scavenge hypochlorous acid more efficiently than ascorbic acid, used as a positive control (IC_50_ = 5.63 ± 0.21 mg/mL).

**Figure 3:**
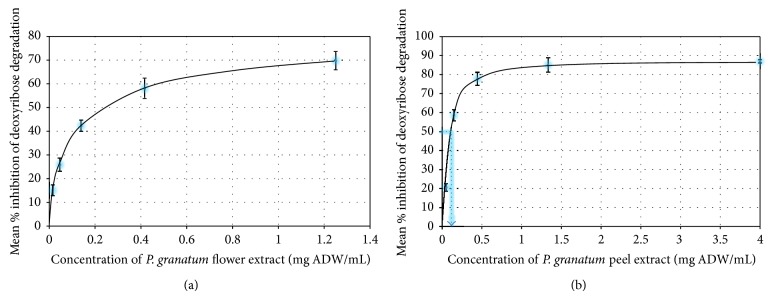
Dose-dependent hydroxyl radical induced deoxyribose degradation inhibition by pomegranate flower and peel extracts. ADW: air dry weight; data are representative of mean ± standard deviation of three replicates. IC_50_ values were extrapolated from the graphs.

The samples analysed were also strong hydroxyl radical scavengers. The results were regarded as indications of hydroxyl radical scavenging propensity by virtue of their ability to inhibit deoxyribose degradation (Figure 3). Pomegranate peel afforded the highest protection, followed by flower, stem, leaf, and seed extracts (Table 2). Statistically significant differences were observed in IC_50_ values among the extracts (*P* < 0.05).

The degree of microsomal lipid peroxidation inhibition induced by Fe^3+^/ascorbate was evaluated by measuring the formation of MDA-(TBA)_2_ adduct spectrophotometrically. All the extracts protected microsome against lipid peroxidation in a dose-dependent manner. Pomegranate flower offered the most prominent protection followed by peel, leaf, and stem extracts. No significant difference was observed among flower, peel, leaf and stem extracts as compared to the seed extract (*P* < 0.05). Gallic acid used as positive control (IC_50_ value of 0.014 ± 0.002 mg/mL) was more potent than the flower extract.

A similar trend was observed for nitric oxide radical inhibition. The flower and peel extracts were the most potent scavenger of NO^•^ with the lowest calculated IC_50_ value (Table 2) and were more effective than ascorbic acid used as positive control (IC_50_ 1253.141 ± 0.002 mg/mL) (*P* < 0.05).

All the extracts exhibited a dose-dependent effect against superoxide radical. However, a different trend of activity for the extracts under study was observed, the stem extract being the most potent followed by leaf, peel, flower, and seed extract. The stem extract was a very powerful scavenger of superoxide (Table 2), 100 folds more powerful than ascorbic acid (IC_50_ 14.191 ± 0.001 mg/mL).

### 3.3. Anti-Inflammatory Effect of *P. granatum* Extracts

**Figure 4:**
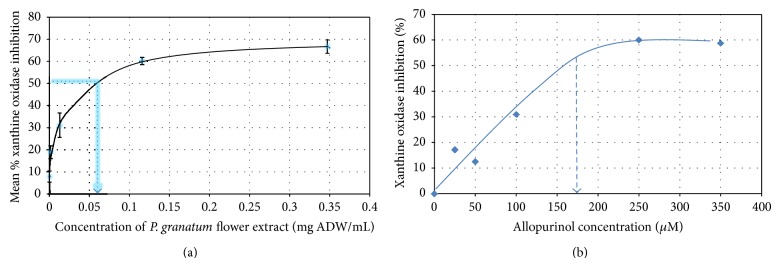
Dose-dependent xanthine oxidase inhibitory activity of pomegranate flower extract and allopurinol. ADW: air dry weight. Data are representative of mean ± standard deviation of three replicates. IC_50_ value was extrapolated from the graph.

The degree of inhibition of xanthine oxidase by the extracts was evaluated by measuring the formation of uric acid spectrophotometrically. Only the flower extract showed xanthine oxidase inhibitory activity, at the concentration range tested. Pomegranate flower extract showed a dose-dependent inhibition of xanthine oxidase with an IC_50_ value of 0.058 ± 0.011 mg ADW/mL (Figure 4). Allopurinol, used as a positive control, was however a more potent inhibitor (IC_50_ value: 0.0055 ± 0.0002 mg/mL).

### 3.4. Antibacterial Activity

The antimicrobial activity of the extracts from *P. granatum* on some indigenous oral microbiotas known to rapidly colonize smooth surfaces and crevices of the teeth and gums causing dental plaque and tooth caries was evaluated. Using the disc diffusion method, it was noted that bacterial growth was minimal in the presence of all concentrated extracts. Peel extract showed greater antibacterial activity and produced the highest inhibition zones against *S. mutans*,* S. mitis*,* and L. acidophilus* (19.75 mm, 25 mm, and 14.75 mm, resp.) (Table 3). This inhibitory effect was observed to be significantly greater than that of the positive control ciprofloxacin (*P* < 0.001). Leaf extract produced the second highest inhibition zones of 16, 18.25, and 8.75 mm against *S. mutans, S. mitis*,* and L. acidophilus* respectively, followed closely by the stem extract. The antibacterial activity effect of the flower extract was much less pronounced compared to its plant counterparts. Although bacterial cell proliferation was minimal, it was still significant compared to the activity of ciprofloxacin (*P* < 0.001).

**Figure 5:**
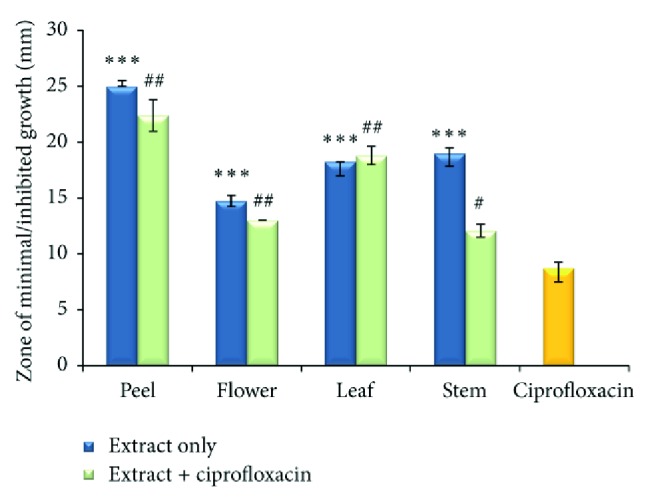
Zone of inhibition or minimum growth (mm) by various parts of *Punica granatum *on* Streptococcus mitis*. Values are expressed as mean where error bars represent standard deviation. Significance compared to ciprofloxacin (positive control): *P* < 0.05^#^, *P* < 0.01^ ##^, *P* < 0.001^***^.

In addition to the study of sole plant extracts, bacterial strains were exposed to a combinational treatment (extract + ciprofloxacin). Such treatment produced inhibition zones that ranged between 12.25 and 19.75 mm against *S. mutans*, 14.75 and 25.0 mm against *S. mitis*, and 8.13 and 11.50 mm against *L. acidophilus*. The following trends were observed: leaf > flower > peel > stem, peel > leaf > flower > stem (Figure 5), and peel < stem < leaf < flower, respectively. For the majority of the plant extracts tested, the combinational treatment proved to exhibit a more efficient antibacterial effect that significantly exceeded that of ciprofloxacin, but not that of individual plant extracts.

## 4. Discussion and Conclusion

In the recent years, *P. granatum* L. has received considerable attention for its pluripharmacological effects and its potent contribution in the maintenance of human health. The efficacy of pomegranate juice has been validated in clinical trials, wherein its ability to decrease inflammatory biomarkers, oxidation of lipids and proteins [9] and to prolong the doubling time of prostate specific antigen in patients with prostate cancer [10] was reported. Phytoconstituents encompassing several phenolic classes [26–28], fatty acids [29], sugars, and organic acids [27, 30, 31] have been characterized in pomegranate fruits and have been ascribed for the diverse pharmacological effects. The edible parts of pomegranate have long been used as food, while the nonedible parts like the roots, rinds, and leaves have a number of applications in ethnomedicine [4, 6]. Thus it can be envisaged that the nonedible parts of pomegranate represent a beneficial source of functional ingredients, a statement warranting in-depth investigations. The present study therefore aimed at determining the phytophenolic content and bioefficacy of the pomegranate plant that has been naturalized in the island of Mauritius since 1639 [7]. Different plant parts, namely, the flower, leaf, stem, peel, and seeds were investigated to assess the *in vitro *prophylactic potential. 

A significant variation in total phenolics concentration was found among the different parts of pomegranate studied. The flower extract contained the highest phenolic level followed by the peel, leaf, and stem while the seed was relatively poor (Table 1). However, Zhang et al. [32] reported that the ethanolic extracts of pomegranate peel extract from China contained higher levels of total phenolics (508.98 ± 24.19 mg gallic acid equivalent/g DW) compared to the flower receptacles (454.96 ± 18.34 mg gallic acid equivalent/g DW) and leaf extracts (289.76 ± 14.82 mg gallic acid equivalent/g DW). Tehranifar et al. [33] also reported higher total phenolics in the methanolic extracts of peel (423.5 ± 31.8 mg gallic acid equivalent/g DW) followed by seed (384.7 ± 24.2 mg gallic acid equivalent/g DW), while the lowest amounts were measured in the leaf extract (133.3 ± 8.7 mg gallic acid equivalent/g DW). 

Using the method of Zhishen et al. [19], the following trend was established for the total flavonoids in the pomegranate extracts: flower > peel > leaf > stem > seed extracts. The HCl/Butan-1-ol assay, on the other hand, indicated low levels of proanthocyanidins in the following order: peel > flower > stem > leaf > seed extracts. 

The TPC measured in this study varied considerably with regard to data from the literature [32, 33]. Factors generally contributing to these variations can include treatment mode of samples prior to extraction; in this study plant parts were air dried, extraction methods and solvents [34] and cultivars used [26]. In addition, phenolic and flavonoid contents have been reported to vary due to seasonal changes and the degree of maturation of the plant parts. For instance, the biosynthesis of flavonols has been documented to be light dependent and can also be affected by temperature variation [35, 36]. Plants growing in Mauritius are tolerant of high level of environment stress induced by varying level of sunlight, ultraviolet radiation, and temperature change throughout the year. This may explain the interesting levels of phenolic compounds in the parts studied. 

A multimethod approach was used to determine the antioxidant effect of the extracts since no one method can predict the total antioxidant efficiency of an extract [17, 37]. Thus, several independent methods differing in biological action mechanisms were used to provide a thorough mechanistic insight of the antioxidant actions of the extracts under study. Nevertheless, a very strong correlation was observed between results of each antioxidant test. For instance, HOCl scavenging activity was highly correlated with deoxyribose assay results (*r* = 0.968, *P* < 0.050). A similar relationship was observed between deoxyribose assay and lipid peroxidation assay, while the superoxide anion radical scavenging activity was significantly and highly correlated with the iron chelating activity (*r* = 0.997, *P* < 0.001), the antioxidant activity from HOCl assay (*r* = 0.999, *P* < 0.001), deoxyribose assay (*r* = 0.964, *P* < 0.01), lipid peroxidation assay (*r* = 0.997, *P* < 0.001), and nitric oxide assay (*r* = 0.927, *P* < 0.050). This is further supported by the very high correlation between the calculated IC_50_ values from iron chelation and the inhibition of microsomal lipid peroxidation assay (*r* = 0.9991, *P* < 0.0001).

The TEAC value provided a ranking order of the antioxidant capacity of the extracts mainly peel > flower > leaf > stem > seed extracts. The TEAC value measured in this study was higher than that reported in the literature; for instance, the TEAC value for peel extract was higher than that reported by Shan et al. [38]. A very strong positive correlation between TEAC and proanthocyanidin content (*r* = 0.921, *P* < 0.05) and TFC was observed (*r* = 0.936, *P* < 0.05).

The extracts under study were also potent scavengers of a number of biologically relevant radicals. The HOCl scavenging assay indicated the peel extract as the most potent scavenger of hypochlorous acid. The antioxidant capacity hierarchy based on the HOCl assay of the extracts was in the following order of activity: peel > flower > leaf > seed extracts, the seed extract showing similar efficacy to ascorbic acid (IC_50_ value 5.63 ± 0.21 mg/mL). Similarly, the peel extract was the strongest inhibitor of deoxyribose sugar degradation against hydroxyl radicals generated via the Fenton reaction (Table 2). Only a moderate correlation between polyphenolic content and IC_50_ values was found. 

The superoxide anion radical scavenging assay showed a different trend in activities compared to other antioxidant systems. Interestingly, the stem extract was found to be more potent than the leaf, flower, and seed extracts. This finding is in line with data reported by Kaneria et al. [34], whereby pomegranate stem extract exhibited higher antioxidant activity than leaf extract in both DPPH antiradical assay and the superoxide anion radical assay. On the other hand, the flower extract also exhibited interesting antioxidant potential. The latter was the most potent inhibitor of nitric oxide followed by peel, stem, leaf, and seed extracts. 

All the pomegranate extracts significantly inhibited Fe^3+^/ascorbate-induced microsomal lipid peroxidation with a calculated IC_50_ of less than 1.7 mg ADW/mL except for the seed extract. The ability of the pomegranate peel extract to inhibit lipid peroxidation of beef liver microsome was consistent with the findings of Althunibat et al. [39] who reported decreased lipid peroxidation in liver and kidney homogenate of STZ-induced diabetic rat models. The lipid protective ability of the extracts may be partly attributed to its flavonoid content (*r* = 0.630, *P* < 0.05). For instance, the O-dihydroxyl groups in the flavonol ring structure have been reported to be a potent inhibitor of lipid peroxidation in cells [40]. 

Complex formation with reduced form of transition metals particularly those that can enhance metal-induced free radical generation has been proposed as an alternative antioxidant mechanism of action ascribed to plant phytophenolic. Flavonoids can act by chelating metal ions thereby inhibiting free radical production [41]. In this line, the iron (II) ions chelating ability of the different extracts was investigated. The hierarchy of metal iron chelation of the plant parts was flower > peel > stem > leaf > seed and paralleled the nitric oxide inhibition (Table 2) (*r* = 0.947, *P* < 0.05). The similarity in activity trend in both assays may be attributed to the involvement of the catechol moiety of the flavonoids as part of the mechanism employed in both assays. The structural requirements and the mechanism of nitric oxide production inhibition by flavonoids have been reported [42]. The metal chelating activity correlated with TFC (*r* = 0.626) which may be partly assigned to the chemical structure of flavonoids. The catechol moiety in the ring B, the 3-hydroxyl and 4- oxo groups in the heterocyclic ring C, and the 4- oxo and 5-hydroxyl groups between the C and A rings has been identified as binding sites for metal ions in the flavonoid molecules [41, 43]. In addition, the vital role of Fe^2+^ in inducing and propagating lipid peroxidation has been well documented in the literature [44, 45], and thus iron chelation can be proposed as a mechanism for the potent inhibition of microsomal lipid peroxidation by the flower and peel extracts. 

Similarly, the FRAP assay based on the redox reaction involving electron transfer showed the following hierarchy of activity: flower > peel > leaf > stem > seed. The FRAP assay does not detect antioxidant compounds that act by hydrogen atom transfer. The FRAP values of this study were consistent with data from Ardekani et al. [26] who reported FRAP value of peel extract to vary between 3401 and 4788 *μ*mol Fe^2+^/g DW among different cultivars. Statistically significant positive correlation was obtained between the FRAP and TPC (*r* = 0.996, *P* < 0.01) as well as with TFC (*r* = 0.983, *P* < 0.01). Numerous reports showed similar types of linear relationship between antioxidant activities and phytophenolic contents of fruits [37, 46]. 

Excess of uric acid in joints has been associated with inflammation [47] leading to pathological conditions. Xanthine oxidase, an important enzyme involved in the conversion of hypoxanthine to xanthine and to uric acid, has been reported as an interesting target against inflammation. In this vein, xanthine oxidase inhibitory activities of the pomegranate plant parts were thus assessed. The pomegranate flower extract exhibited a dose-dependent enzyme inhibition propensity. However, the inhibitory potential of the flower extract as measured by the calculated IC_50_ (0.058 ± 0.011 mg ADW/mL) was weaker than allopurinol (IC_50_: 0.0055 ± 0.0002 mg/mL) used as control. Other parts extracts showed no inhibitory activity at the tested concentration. 

The extracts tested behaved differently in the various experimental systems showing varying hierarchy of activities which were independent of the phenolic content measured. The general trend in bioefficacy demonstrated that pomegranate flower and peel extracts were very potent followed by leaf > stem > seed extracts. The findings indicated that polyphenolic compounds may act synergistically to potentiate the antioxidant activity of extracts. It should also be noted that the pomegranate flower and peel extracts were 100 times more potent than the red and yellow *Psidium cattleianum* Sabine “Chinese guava,” Mauritian exotic fruits evaluated using the TEAC and FRAP assays [48]. In addition, pomegranate flower and peel extracts were found to be much more potent than the citrus extracts assayed using similar methodology. For instance, the total phenolic content of pomegranate flower extract was 200 folds higher than *Fortunella margarita* pulp extract (1694 ± 19 µg/g FW) [50] and 44 folds higher than *C. reticulata* X *C. sinensis* flavendo extract (7667 ± 93 µg/g FW) [38]. Similarly, the total flavonoid content were 200 folds higher than *Citrus maxima* pulp extract (965 ± 7 µg/g FW) [50] and 35 folds higher than *C. reticulata* X *C. paradisi* flavendo extract (5615 ± 93 µg/g FW) [38]. Likewise, the antioxidant propensities of pomegranate flower and peel extracts were significantly more important than the Mauritian citrus fruits pulp of flavendo extract thereby highlighting the prophylactic potential of the extracts under study. 

Furthermore, the growing prevalence of dental caries, gingivitis, periodontitis, and oral microbial infections cases amongst adults prompted the evaluation of the antibacterial effects of the extracts against oral bacterial growth. Pathophysiological mechanisms including deficient nutritional intake, alterations in host response to oral microflora, compromised neutrophil function, and decreased phagocytosis and leukotaxis have been increasingly suggested to account for these disorders. A realistic management plan including regular oral hygiene practice and basic dental treatment can be envisaged for managing dental caries and its associated oral complications. Nowadays, active constituents extracted from plants have been included in the preparation of toothpaste, mouth rinses, dental floss, and chewing gum to ensure a stronger antimicrobial activity [8]. Ongoing studies focusing on the anticariogenic properties of polyphenols isolated from green tea [50], cranberry juice [51], and shiitake mushrooms [52] seemed promising. However despite the numerous studies conducted on such functional foods, only a handful of them can be clinically used to control dental plaque, caries formation, and mouth infections due to their effectiveness, stability, taste, and economic feasibility [53]. George and Sumathy [54] reported effective antibacterial activity of aqueous and ethanolic extracts of pomegranate against *Streptococcus mutans, Staphylococcus *sp.,* Escherichia coli, Lactobacillus *sps., and* Candida albicans* isolated from the mouth. In this study, pomegranate was reported to negatively influence the proliferation of Gram Positive bacteria and concurrently to demonstrate potent iron-chelating capabilities. Recently, Kulkarni et al. [55] found that punicalagin, an ellagitannin isolated from the pomegranate peel, could completely suppress iron catalyzing oxidant reactions *in vitro. *It can therefore be speculated that potent pomegranate extracts under study, in particular, the peel extract, may be liable to remove iron from the broth medium and deprive bacteria of the iron they need for normal growth through chelation in view of the high iron (II) ions chelating efficiency. The presence of both tannins and alkaloids isolated from pomegranate pericarp and seeds has been extensively reviewed for their outstanding ability to block bacterial surface adhesions and inhibit glycosyltransferases thus deterring bacterial attachment to dental surfaces, hence, its colonization [56]. Data from this study provide basic supplementary evidence of the antimicrobial activity of pomegranate and support the imperative need to find new effective bioagents that can avoid a negative impact upon the future oral health of communities affected by dental caries and expenditure on dental services.

While pomegranate' edible parts have common applications in the food and food processing industries due to their excellent nutritional and health values [57], this study showed the prospect of the nonedible parts Mauritian cultivar of pomegranate. An in-depth comparison of the broad classes of phytophenolic and the bioefficacies of the latter indicated the effectiveness of the flower and peel extracts. The available evidence indicates that these extracts might be of therapeutic benefit in bacterial infections and be an ideal candidate for functional food health products. Further investigations need to be directed towards determining the potential toxicity, the phytochemistry of the nonedible parts, and applicability of the extracts in various food matrices. The use of functional foods enriched with pomegranate flower and peel extracts however needs technologies for incorporating these health-promoting ingredients into food without reducing their bioavailability or functionality.

## Figures and Tables

**Table 1:** Total phenolics, total flavonoids, and total proanthocyanidin content of pomegranate parts extracts.

	TPC mg GAE/g ADW ± SD	TFC mg QE/g ADW ± SD	TPrC mg CCE/g ADW ± SD
Flower	336.51 ± 0.70^a^	213.54 ± 3.14^a^	1.46 ± 0.06^a,b^
Peel	190.27 ± 0.54^b^	180.10 ± 1.31^b^	2.48 ± 0.08^a^
Leaf	87.81 ± 0.47^c^	63.89 ± 0.62^c^	0.21 ± 0.01^b^
Stem	52.92 ± 0.62^d^	41.36 ± 0.52^d^	0.32 ± 0.01^b^
Seed	0.65 ± 0.00^e^	0.33 ± 0.00^e^	0.13 ± 0.00^b^
LSD value at 5% significance	0.96	2.85	1.29

ADW: air dry weight; CCE: cyanidin chloride equivalent; GAE: gallic acid equivalent; QE: quercetin equivalent; TPC: total phenolic content; TFC: Total Flavonoid content; TPrC: Total proanthocyanidin content. Different superscripts between rows in individual columns represent significant difference between extracts. Data expressed as mean ± standard deviation (*n* = 3).

**Table 2:** Antioxidant activities of different pomegranate parts extract.

Extract	Fe(II) chelating activity	HOCl scavenging activity	Inhibition of deoxyribose degradation	Inhibition of lipid peroxidation	Nitric oxide inhibition	Superoxide scavenging
Flower	0.113 ± 0.006^c^	0.012 ± 0.001^b^	0.220 ± 0.041^c^	0.047 ± 0.006^b^	0.396 ± 0.002^e^	0.175 ± 0.001^b^
Peel	0.157 ± 0.006^c^	0.004 ± 0.001^b^	0.111 ± 0.001^c^	0.333 ± 0.058^b^	0.668 ± 0.001^d^	0.089 ± 0.001^c^
Leaf	0.713 ± 0.006^b^	0.017 ± 0.002^b^	3.752 ± 0.091^b^	0.601 ± 0.100^b^	18.155 ± 0.005^b^	0.072 ± 0.001^d^
Stem	0.397 ± 0.030^c^	N.A	0.480 ± 0.031^c^	1.700 ± 0.173^b^	4.831 ± 0.001^c^	0.040 ± 0.001^e^
Seed	15.400 ± 0.310^a^	5.200 ± 0.400^a^	14.300 ± 0.760^a^	35.000 ± 2.646^a^	48.641 ± 0.001^a^	2.523 ± 0.001^a^
LSD value at 5% significance	0.300	0.371	0.630	2.162	3.231	1.490

N.A: not available. Data expressed as mean IC_50_ ± standard deviation (mg/mL) (*n* = 3); different superscripts between rows in individual columns represent significant difference between extracts.

**Table 3:** Zone of inhibition or minimum growth (mm) by various parts of *Punica granatum *on *S. mutans, S. mitis*, and *L. acidophilus*.

	Mean zone of inhibition ± SD (mm)		
	*Streptococcus mutans *	*Streptococcus mitis *	*Lactobacillus acidophilus *
Extract only			
Peel	19.75 ± 0.50^***^	25.00 ± 0.00^***^	14.75 ± 2.22^*^
Flower	12.25 ± 0.50^*^	14.75 ± 0.50^***^	8.38 ± 0.75
Leaf	16.00 ± 0.00^***^	18.25 ± 0.50^***^	8.75 ± 0.50
Stem	14.75 ± 0.50^***^	19.00 ± 1.15^***^	8.75 ± 0.50
Extract + ciprofloxacin			
Peel	10.75 ± 0.96	22.41 ± 1.41^##^	11.50 ± 1.00^#^
Flower	11.25 ± 0.96	13.00 ± 0.00^##^	8.13 ± 1.03
Leaf	12.50 ± 0.58^##^	18.82 ± 0.82^##^	8.25 ± 0.50
Stem	10.0 ± 0.0	12.08 ± 0.58^#^	9.25 ± 1.26
Ciprofloxacin	10.25 ± 0.50	8.75 ± 1.26	8.75 ± 0.50

Values are expressed as mean ± standard deviation. Significance compared to ciprofloxacin (positive control): *P* < 0.05^∗,#^, *P* < 0.01^##^, *P* < 0.001^***^.
